# Inline monitoring of high cell density cultivation of *Scenedesmus rubescens* in a mesh ultra-thin layer photobioreactor by photon density wave spectroscopy

**DOI:** 10.1186/s13104-022-05943-2

**Published:** 2022-02-15

**Authors:** Michael Sandmann, Marvin Münzberg, Lena Bressel, Oliver Reich, Roland Hass

**Affiliations:** 1 University of Applied Sciences Neubrandenburg, Brodaer Straße 2, 17033 Neubrandenburg, Germany; 2 Physical Chemistry-innoFSPEC Potsdam, Institute of Chemistry, University of Potsdam, Am Mühlenberg 3, 14476 Potsdam, Germany; 3 PDW Analytics GmbH, Geiselbergstr. 4, 14476 Potsdam, Germany

**Keywords:** Photon density wave spectroscopy, Multiple light scattering, Process analytical technology, Fiber-optical spectroscopy, Mesh ultra-thin layer photobioreactor

## Abstract

**Objective:**

Due to multiple light scattering that occurs inside and between cells, quantitative optical spectroscopy in turbid biological suspensions is still a major challenge. This includes also optical inline determination of biomass in bioprocessing. Photon Density Wave (PDW) spectroscopy, a technique based on multiple light scattering, enables the independent and absolute determination of optical key parameters of concentrated cell suspensions, which allow to determine biomass during cultivation.

**Results:**

A unique reactor type, called “mesh ultra-thin layer photobioreactor” was used to create a highly concentrated algal suspension. PDW spectroscopy measurements were carried out continuously in the reactor without any need of sampling or sample preparation, over 3 weeks, and with 10-min time resolution. Conventional dry matter content and coulter counter measurements have been employed as established offline reference analysis. The PBR allowed peak cell dry weight (CDW) of 33.4 g L^−1^. It is shown that the reduced scattering coefficient determined by PDW spectroscopy is strongly correlated with the biomass concentration in suspension and is thus suitable for process understanding. The reactor in combination with the fiber-optical measurement approach will lead to a better process management.

**Supplementary Information:**

The online version contains supplementary material available at 10.1186/s13104-022-05943-2.

## Introduction

Algae are one of the most promising candidates for solving the problem of nowadays need for renewable energy and sustainable food [1–7]. In comparison to heterotrophic production systems like yeasts, phototrophic production of algae still needs significant improvement to operate cultivations at high cell densities. To close this gap, different types of photobioreactors (PBR) were devised in the past but up-scaling into an industrial scale is still a challenge and the initial investment and operating costs are still high [8]. The optimization of PBR in terms of light harvest, nutrient supply, or gas exchange is of utmost importance for improvement of the biomass production efficiency.

One potential approach to monitor the outcome of PBR design-changes, is the implementation of suitable inline process analytical technologies (PAT), providing access to algal growth kinetics also at very high cell concentrations. A common approach is to monitor the process state either by dry matter content determination as established offline analysis or by optical density probes [9–11]. Offline analyses use aliquots of the cell culture which often increases the risk of contamination with other microbes, is time consuming and thus gives strongly delayed process information. Due to multiple light scattering that occurs inside and between cells, quantitative optical inline determination of biomass by, e.g., optical density probes, is still a major challenge [11]. In this study, an advanced optical method, called Photon Density Wave (PDW) spectroscopy was applied as inline tool to evaluate if monitoring of algal growth in a high cell density cultivation is possible and to which extend it reflects results of accepted reference techniques. PDW spectroscopy, a technique based on multiple light scattering, enables the independent and absolute determination of optical key parameters of concentrated suspensions [12]. It has been recently applied to several chemical, physical, and biotechnological processes, but has not yet been implemented in algae cultivation [13–19]. High algal cell concentrations have been obtained by a so-called “mesh ultra-thin layer (MUTL) PBR”. The unique reactor design was recently described [20–22]. However, detailed knowledge about, e.g., multiphase-fluid dynamics, light penetration, or effects of the specialized geometry on algal biology is still missing. Here, this PBR was used to generate a high cell density cultivation with a CDW of 33.4 g L^−1^. PDW spectroscopy was used to investigate biomass dynamics over time in this novel type of PBR. Aim of this study is the investigation of the suitability of PDW spectroscopy for inline biomass monitoring during high cell density cultivations of algae.

## Main text

### Experimental section

#### Photobioreactor

**Fig. 1:**
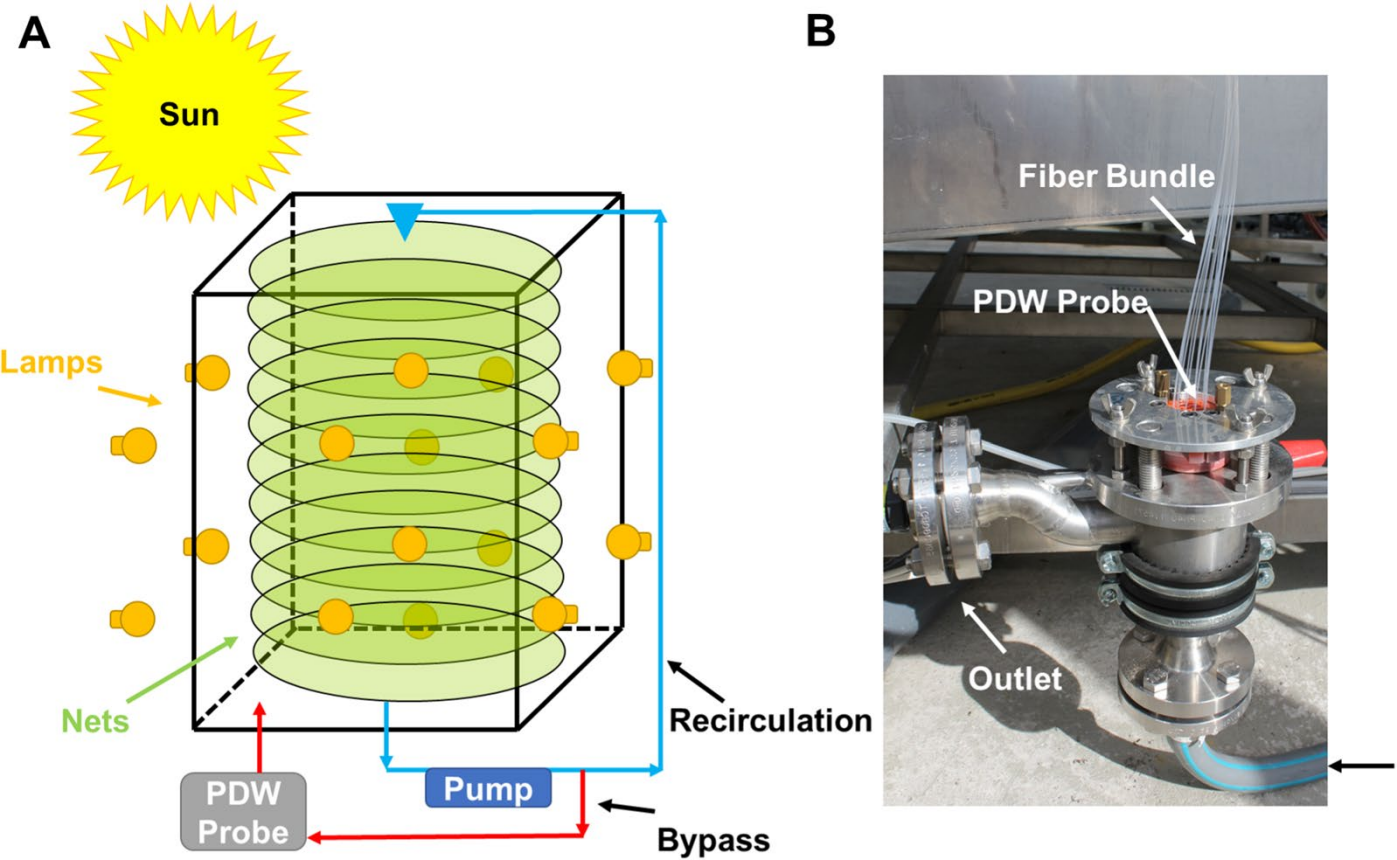
Experimental setup. **A** Schematic drawing of the MUTL PBR including a PDW spectroscopy probe. **B** Photograph of the PDW probe mounted on the MUTL PBR recirculation loop

Algal cultivation was performed under greenhouse conditions in a prototype MUTL PBR at the IGV GmbH (Nuthetal, Germany). The experiment has been executed during August and September in 2012. The greenhouse was used as transparent shell to protect a collection of different PBR against harsh weather conditions and to prolong the growth period of the algae. Different to the MUTL PBR, the greenhouse itself was not illuminated or temperature controlled. A scheme of the PBR is displayed in Fig. 1A and the adapter for the PDW spectroscopy process probe is shown in Fig. 1B.

Briefly, the configuration of the PBR used in this work consists of 40 vertically stacked horizontal polymer nets with an average distance of 0.05 m between them. The polymer nets are enclosed in a 2.5 m high, transparent polymethyl methacrylate housing, occupying a footprint area of 4.84 m^2^. The water-cooled double bottom of the reactor is coupled to an external cooling device. The PBR is equipped with a continuously working recirculation loop to pump algal suspension from the sink to the top of the PBR from which the suspension is sprayed by spiral jet nozzles on the polymer nets. Such nozzles need a relatively high liquid pressure of 1 to 3 bar but have a high clog-resistance [22]. During their movement through the stack of nets, cells are exposed to the incident light and the surrounding gas phase. The PDW spectroscopy probe is inserted in a special flow-cell implemented in the recirculation loop (Fig. 1B). Nitrate content was measured during algal growth with the ‘Nitrate Cell Test-Kit in seawater’ from WTW (Xylem Analytics Germany Sales GmbH & Co. KG, WTW, Weilheim, Germany) and a concentrated solution of all five macronutrients were added if around 40% of the starting concentration of nitrate was reached during the experiment. This procedure was based on the assumption that all macro constituents will deplete in parallel. During the cultivation, biomass growth was supported through additional 16 high pressure sodium lamps with 400 W power each. The lamps have been distributed on all four sides of the PBR. The growth experiment was executed for 21 days.

#### Culturing of *Scenedesmus rubescence*

*Scenedesmus rubescence* strain SAG 5.95 was obtained from the SAG Culture Collection of Algae (Göttingen, Germany). The preculturing was done in ten lab-scale bubble columns (1.8 L suspension volume each) under continuous light with a light intensity of 120 µmol photons m^−2^ s^−1^ at 25 °C and 3% CO_2_ [v/v] in a synthetic growth media called ½ Tamiya [23, 24]. Further up-scaling for production of the inoculum culture for the cultivation experiment within the MUTL-PBR was done in a 90 L tubular PBR with the same growth media and an initial CDW of 0.3 g L^−1^. The 90 L PBR was already located in the same greenhouse as the MUTL-PBR. The cells have been prevented from heat-shock by an automated water-cooling by sprinkling. After reaching the beginning of stationary growth phase a part of the cell suspension from the 90 L PBR was used to inoculate the MUTL-PBR. Pre-culture was performed under axenic conditions. In the further up-scaling and the MUTL experiment this cannot be guaranteed anymore. To keep microbial contaminations on a minimal level all large-scale PBR have been thoroughly cleaned with aqueous sodium peroxide solution [22].

#### Quantification of cell size and cell concentration

Cell size and cell concentration were determined by offline measurements (MULTISIZER 3, Beckman Coulter, Krefeld, Germany) once or twice per day [25, 26]. An important parameter that can be measured by this kind of device is the total cellular volume [(given in cubic micrometers (µm^3^) per milliliter of suspension)]. It is defined as the sum of all cellular volumes present in 1 mL of suspension, is a measure of the biomass or wet-weight concentration of the cells present in suspension and has been widely applied in other studies [27, 28].

#### Determination of the cell dry weight

Determination of CDW was done gravimetrically once or twice per day. Briefly, 5 to 10 mL aliquots from the cell suspension were centrifuged at 3000×*g* for 10 min in pre-weighed glass tubes. The pellet was afterwards washed with deionized water and then dried at 105 °C for 24 h. Prior to the weight measurements, the hot glass tubes were cooled to room temperature in a desiccator. The weight difference corresponds to the dry matter content of the cell suspension.

#### Photon density wave spectroscopy

A PDW spectrometer features an intensity modulated laser as light source. By an optical emission fiber, the laser light is guided into a fiber-optical probe being implemented in a special flow cell [29] in the recirculation loop. The fiber end acts as point-like light source. Due to multiple light scattering and absorption a PDW is created within the algae suspension. Further detection fibers in the probe collect light from the PDW and guide it back to the detector inside the spectrometer. Changes to phase and amplitude of the PDW are characterized by a vector network analyser within the spectrometer. These changes give access to the optical coefficients of the algae suspension.

Here, a self-constructed PDW spectrometer (commercial versions available by PDW Analytics GmbH, Potsdam, Germany) was applied [29] and a measurement wavelength of 906 nm with modulation frequencies from 10 to 810 MHz was used. To characterize the PDW as function of distance between emission and detection fiber, a fiber-optical probe consisting of one central emission fiber and 12 surrounding detection fibers was constructed [29]. The distances ranged from 9.47 mm to 20.38 mm. For raw data analysis, a refractive index of *n* = 1.3264 at 906 nm was used (refractive index of water, obtained as described in [13]). PDW spectroscopy measurements were carried out continuously with 10-min time resolution.

## Results

### Results of the reference analysis

**Fig. 2:**
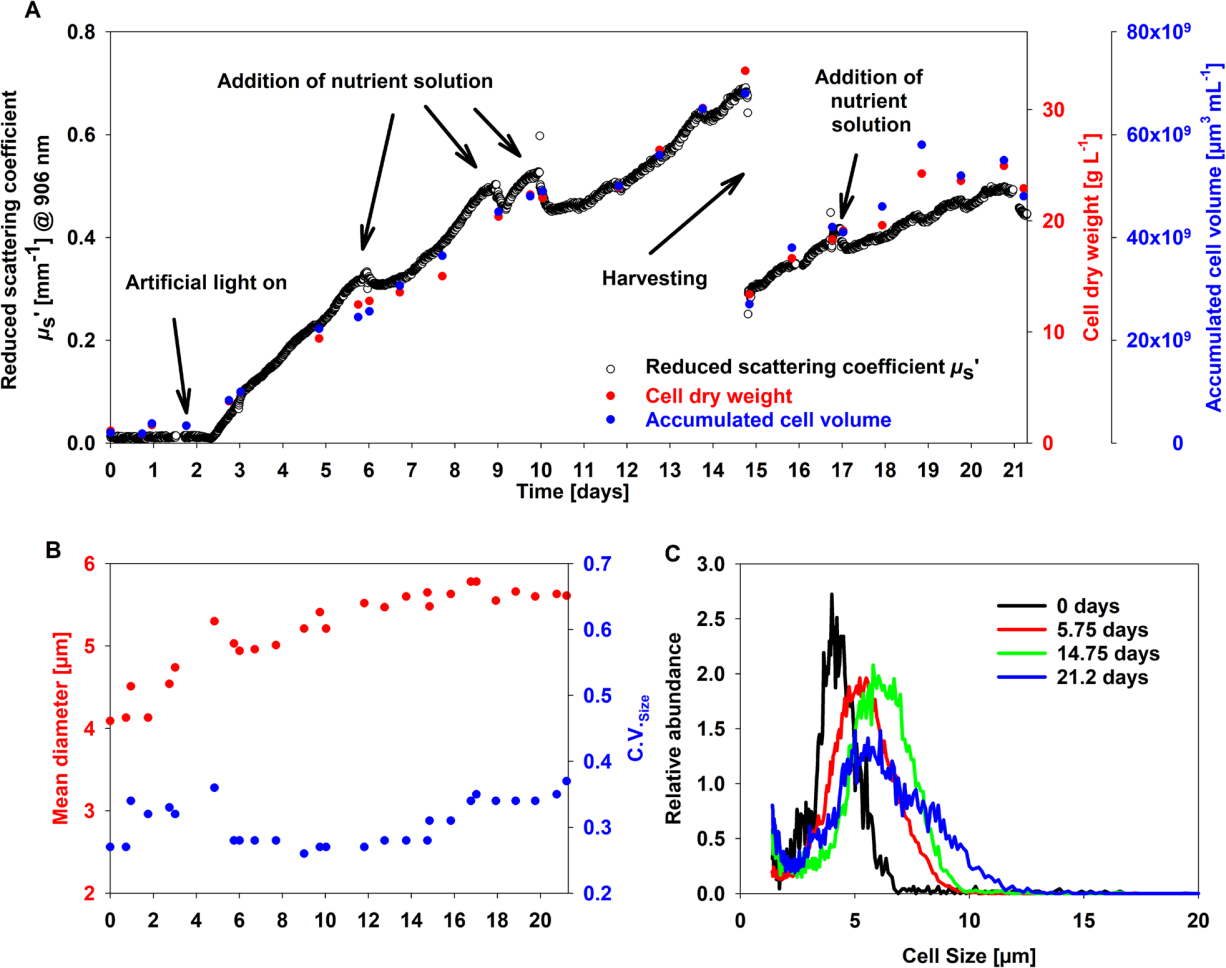
Culture characterization over time. **A** Development of biomass related parameters. **B** Mean diameter and coefficient of variation (CV) as a measure for the width of the cell size distribution. **C** Selected cell size distributions determined by Coulter counter (sample time in legend)

Development of the algal culture in the MUTL PBR is shown in Fig. 2A. Algal cells showed a lack phase of 2 to 3 days and afterwards started strong biomass growth with nearly linear increase of dry matter content in the cell suspension. Close to the end of the lack phase the artificial light was continuously switched on for the rest of the experiment (Fig. 2A). Mean growth rate was comparatively high (2.39 g L^−1^ day^−1^) during first 14 days of growth. At day 14 the cell suspension was partly harvested and refilled with fresh culture media (Fig. 2A) and afterwards the reactor was running for additional 6 days. At different timepoints, concentrated solution of macro nutrients was added to the cell culture (Fig. 2A). The analysis of total cellular volume has been used as additional reference technique. It exhibits a very similar development like the dry matter content. Based on Coulter counter measurements, a deeper analysis of the population dynamics of the algal cells can be obtained (Fig. 2B). During cultivation, cells increased their mean diameter from approximately 4.0 to 5.6 µm (Fig. 2B). Also, the coefficient of variation (CV_Size_) of the cell size is displayed. The CV_Size_ is a relative measure for the widths of a distribution and can be used to quantify the cell-to-cell heterogeneity inside a cell population. The CV_Size_ increased from 0.27 to 0.37, which means an increased heterogeneity. This is visible also in the cell size distributions (Fig. 2C). Cell size ranged between 2 to 12 µm in diameter, which is similar to other Scenedesmus species [24].

### Results of PDW spectroscopy and correlation with the reference analysis

**Fig. 3:**
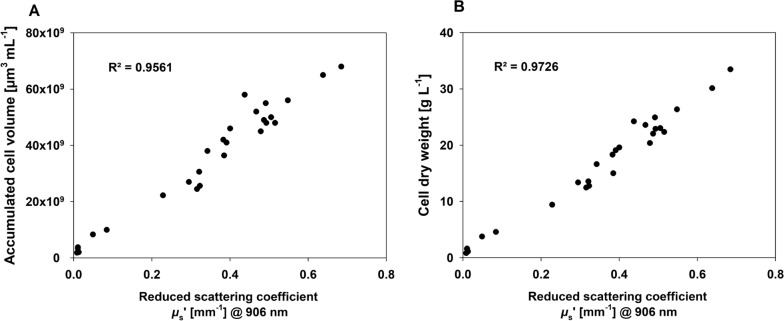
Correlation of PDW spectroscopy with reference analyses. **A** Correlation of *µ*_s_ʹ at 906 nm from PDW spectroscopy with accumulated cellular volume derived from Coulter counter measurements. **B** Correlation of *µ*_s_ʹ at 906 nm from PDW spectroscopy with CDW

PDW spectroscopy determined the optical coefficients with high temporal resolution, unattended, and without sampling. Generally, *µ*_s_ʹ at 906 nm indicates the same dynamics as function of time as both reference analyses (Fig. 2A). Based on *µ*_s_ʹ, dilution effects from the addition of concentrated media component solution and the harvesting procedure can be visualised with high time resolution and even growth rates are obtained in nearly real-time (Additional file 1: Figure S1). However, at low CDW PDW spectroscopy is not able to provide reliable optical coefficients due to too low turbidity of the algae suspension. *µ*_s_ʹ values have been correlated with both corresponding reference analyses, indicating a linear correlation with R^2^ = 0.9726 against CDW and R^2^ = 0.9561 against Coulter counter data (Fig. 3). Correlation of both reference analyses resulted in R^2^ = 0.9895 (Additional file 1: Figure S2).

## Discussion

Quantitative optical inline spectroscopy is still a challenge especially in concentrated biotechnical process [30]. One reason is a parallel occurrence of light absorption and multiple light scattering. The aim of the recent work was to investigate if PDW spectroscopy is applicable as inline tool for monitoring of algal growth in a high cell density cultivation. As parameter of relevance, the reduced scattering coefficient should correlate with different offline reference methods. Photoautotrophic growth of algal cells has been investigated in a very specialized photobioreactor called MUTL (Fig. 1). In comparison to classical PBRs, MUTL-PBRs exhibit significantly higher peak CDW and growth rates. This was shown in three different references, but with a strong spread of the exactly reached peak CDW ranging from 6.6 to 40 g L^−1^ [20–22]. In this study, the MUTL-PBR was used to culture high cell densities up to 33.4 g L^−1^. Conventional offline techniques have been used to describe growth characteristics of the culture. For comparison, inline measurements by PDW spectroscopy were performed directly in the cell culture. PDW spectroscopy provided reduced scattering coefficients (*μ*_s_ʹ) of the algal suspension, with peak values of approx. 0.7 mm^−1^ at 906 nm. In this experiment, *μ*_s_ʹ is found to be strongly correlating with both applied reference analyses (Figs. 2 and 3). PDW spectroscopy was able to measure fast dynamics, e.g., harvesting of the cells and influences of nutrient addition. In comparison to offline analyses, PDW spectroscopy was able to continuously monitor fast changes in the cell suspension, without any need for sampling or sample preparation and with high temporal resolution. It has been shown that also growth rates derived from *μ*_s_ʹ can be calculated (Additional file 1: Figure S1). As presented, the addition of the nutrient solution was based on offline measurements of the nitrate content within the media. The measurements were done only once or twice per day, are labor-intensive and result in a nutrient feed with time delay. A feed strategy that will be directly coupled with the continuously measured growth rate by PDW should be used to establish a real-time adjustment of nutrient feeding. This might result in even better growth rates of the MUTL PBR and should be proven in future experiments. Putative adverse effects that might interfere with the PDW measurements are minor. This was shown through the thorough comparison of the PDW signals with two independent and accepted reference analyses throughout the complex growth trajectory of the cell culture. The comparison resulted in coefficient of determination (R^2^) close to 1 (Fig. 3).

Implementing such a highly advanced fiber-optical technology was never executed before in a PBR. Additionally, up to now the recent work included the most detailed analysis of the trajectory of algal growth in the novel MUTL PBR type. In the past it has been shown that PDW spectroscopy was able to monitor dynamics in processes in much more concentrated suspensions or dispersions. Examples are, e.g., concentrated nanoparticle suspensions with *μ*_s_ʹ of up to 20 mm^−1^ or emulsification processes with *μ*_s_ʹ of more than 5 mm^−1^ [13]. In a biotechnological production of polyhydroxyalkanoate (PHA), CDW of 40 g L^−1^ have been reached corresponding to *μ*_s_ʹ of 2 mm^−1^ [19]. It can be concluded that the reduced scattering coefficient as determined by PDW spectroscopy seems to be a suitable measure for CDW in diverse biotechnological processes, including high cell density algal growth. Additionally, *μ*_s_ʹ can be used to calculate growth rates in nearly real-time, enabling process monitoring even in highly concentrated suspensions. Furthermore, the determined optical parameters of algal cells can be used in future approaches to model light penetration within PBRs which is expected to be a key to enhance reactor performance [31, 32]. Thus, there is a high potential for PDW spectroscopy to be used for advanced process monitoring, process control, and to ensure a better process understanding in concentrated biotechnological suspensions.

## Limitations

- Data from reference analysis were taken without replicates.
- Cultivation reproducibility with respect to CDW of 33.4 g L^−1^ was not tested. However, focus here was on suitability of PDW spectroscopy for inline biomass monitoring.
- Raw data analysis for PDW spectroscopy took assumptions into account, referring to the refractive index and volume fraction of cells, respectively.
- The flow cell for PDW spectroscopy probe implementation was not optimized for reduction of shear forces onto cells. Thus, structural damage might have been induced.

## Supplementary Information

**Additional file 1: Figure S1.** Growth rate (GR_PDW,in-line_) of the algal cells based on µ_s_ʹ. Growth rate is based on the slope within the time increments (*µ*_s_ʹ/time). Original data from *µ*_s_ʹ was smoothed before analysis with a simple moving average based on 31 data points. Plotting of growth rate is done with simple moving average based on 11 data points. *µ*_s_ʹ is plotted as unsmoothed data as given in figure 2 A. **Figure S2.** Correlation of both reference analyses.

## Data Availability

The original datasets and other miscellaneous materials related to the investigation are available upon reasonable request to the corresponding author.

## Abbreviations

PDW: Photon density wave
CDW: Cell dry weight
MUTL: Mesh ultra-thin layer
PAT: Process analytical technology
PBR: Photobioreactor
Min: Minute
CV: Coefficient of variation
GR_PDW,inline_: Growth rate based on *µ*_*s*_
