# Supplementary material for: Inline monitoring of high cell density cultivation of Scenedesmus rubescens in a mesh ultra-thin layer photobioreactor by photon density wave spectroscopy
Source: BMC Res Notes. 2022 Feb 15;15:54. doi: 10.1186/s13104-022-05943-2 (PMC8845379; doi:10.1186/s13104-022-05943-2)
Supplement: Supplementary file 1 — Additional file 1: Figure S1. Growth rate (GRPDW,in-line) of the algal cells based on µsʹ. Growth rate is based on the slope within the time increments (µsʹ/time). Original data from µsʹ was smoothed before analysis with a simple moving average based on 31 data points. Plotting of growth rate is done with simple moving average based on 11 data points. µsʹ is plotted as unsmoothed data as given in figure 2 A. Figure S2. Correlation of both reference analyses. [file 13104_2022_5943_MOESM1_ESM.docx]

Additional file 1 Material

Michael Sandmann*^1^*, Marvin Münzberg^2^, Lena Bressel^2^, Oliver Reich², and Roland Hass^2,3^

^1#^University of Applied Sciences Neubrandenburg, Brodaer Straße 2, D-17033 Neubrandenburg, Germany

^2^University of Potsdam, Institute of Chemistry, Physical Chemistry – innoFSPEC Potsdam, Am Mühlenberg 3, D-14476 Potsdam, Germany

^3^PDW Analytics GmbH, Geiselbergstr. 4, 14476 Potsdam, Germany

^#^sandmann@hs-nb.de





**Figure S1: Growth rate (GR_PDW,in-line_) of the algal cells based on µ_s_’.** Growth rate is based on the slope within the time increments (*µ*_s_’ / time). Original data from *µ*_s_’ was smoothed before analysis with a simple moving average based on 31 data points. Plotting of growth rate is done with simple moving average based on 11 data points. *µ*_s_’ is plotted as unsmoothed data as given in figure 2 A.





**Figure S2: Correlation of both reference analyses.**
